# Postural stability and body composition according to primary dysmenorrhea severity in young women: a cross-sectional study

**DOI:** 10.3389/fendo.2026.1919973

**Published:** 2026-09-17

**Authors:** Paulina Kruczek, Grzegorz Przysada, Maciej Tomasz Kochman

**Affiliations:** 1 Faculty of Health Sciences and Psychology, University of Rzeszów, Rzeszów, Poland; 2 Faculty of Medicine, University of Rzeszów, Rzeszów, Poland; 3 University Center for Research and Development in Health Sciences, University of Rzeszów, Rzeszów, Poland

**Keywords:** bioelectrical impedance analysis, hormonal fluctuations, menstrual cycle, physical performance, postural control, primary dysmenorrhea, women’s health

## Abstract

**Background/objectives:**

Primary dysmenorrhea (PD) is a common but often underestimated problem among women that may influence sensory processing and neuromuscular control. However, the associations between menstrual pain intensity, body composition, and postural stability remain unclear. Understanding whether menstrual pain is associated with measurable changes in neuromuscular performance may provide insight into female-specific determinants of physical function.

**Methods:**

This cross-sectional study included 99 women aged 18–28 years. The participants were divided into one of three groups based on a retrospective assessment of menstrual pain intensity using the VAS scale (mild, moderate, and severe pain). Static postural stability was assessed using the Neurocom system with the modified Clinical Test of Sensory Interaction on Balance protocol (mCTSIB). Body composition was assessed using bioelectrical impedance analysis (BIA).

**Results:**

No significant differences were observed in static balance or body composition parameters between groups with different PD severity. However, correlation analysis revealed a significant negative association between pain intensity and sway velocity during the firm surface eyes-closed condition in the mild PD group (r_s_ = -0.71; p<0.001), whereas similar relationships were not observed in moderate or severe PD groups. Given the small subgroup size, this correlation should be considered exploratory and interpreted with caution.

**Conclusions:**

The severity of primary dysmenorrhea was not associated with differences in overall static postural stability or body composition. Nevertheless, individual pain-related adaptations in postural control may occur, highlighting the importance of individualized assessment and management strategies in women with PD.

## 1 Introduction

Primary Dysmenorrhea (PD) is a highly prevalent condition among women of reproductive age, with reported prevalence rates ranging from 24% to 92% (1). It carries a substantial socioeconomic burden, acting as a primary driver of school and workplace absenteeism among young females, which significantly impairs their daily functioning and academic or professional productivity (2). Despite its significant impact on quality of life, the true prevalence of dysmenorrhea remains underestimated, largely due to the common tendency among patients to underreport symptoms and refrain from seeking medical consultation (3).

Although the exact pathophysiology of primary dysmenorrhea remains to be fully elucidated, current literature highlights the pivotal role of enhanced prostaglandin F2α and E2 synthesis during endometrial shedding (4). The abrupt decline in progesterone levels at the end of the luteal phase triggers the release of lysosomal enzymes and the subsequent activation of the arachidonic acid cascade (5, 6). Consequently, this cascade induces severe uterine hypercontractility. This elevated myometrial activity generates an intrauterine pressure that exceeds local arterial pressure, directly leading to acute tissue ischemia and pelvic hypoxia (7). The resulting accumulation of anaerobic metabolic byproducts stimulates unmyelinated C-fiber nociceptors, clinically manifesting as painful cramping and menstrual pain symptoms (8, 9).

Growing evidence suggests that the effect of PD may extend beyond the pelvis and involve broader neuromuscular and sensory processing mechanisms. Although the underlying mechanisms remain under investigation, recent research has highlighted the involvement of complex pain-related mechanisms in women with PD (10, 11). Features consistent with central sensitization have also been reported in a substantial proportion of women with PD (11).

Some studies have reported associations between PD and selected postural deviations (12, 13). Although the clinical significance of these findings remains unclear, altered posture may potentially influence postural control mechanisms (14).

While Body Mass Index (BMI) has been reported as a potential factor associated with primary dysmenorrhea (15), reliance on BMI alone may limit the depth of epidemiological investigation. Importantly, BMI does not consistently reflect body composition, as it fails to distinguish between lean mass and adipose tissue. A key argument for expanding research to include body composition analysis in women with PD is the phenomenon of ‘normal-weight obesity.’ This condition is characterized by a BMI within the normal range alongside a high body fat percentage and is estimated to affect approximately 30% of young women in some populations (16–18). Therefore, direct assessment of body composition may provide additional information rather than BMI.

Adipose tissue contributes to systemic inflammatory activity through the secretion of adipokines and inflammatory mediators. Therefore, body composition may represent a biologically relevant factor in the context of PD and its clinical manifestations (19).

Maintaining postural stability requires the precise integration of visual, vestibular, and proprioceptive inputs. However, chronic pain conditions, such as PD, may influence sensorimotor processing and, consequently, postural control (20–22). Therefore, balance assessment under varying sensory conditions may provide insight into potential alterations in postural control associated with PD.

The existing literature provides limited evidence regarding the specific role of body composition components on PD. Furthermore, studies investigating the relationship between body composition, postural stability, and menstrual pain intensity remain scarce and are frequently characterized by small sample sizes. Consequently, there is a lack of definitive findings to guide preventive and therapeutic strategies for young women suffering from PD. Although previous studies have investigated either menstrual symptoms, body composition, or balance performance separately, relatively few studies have examined the relationships among these factors in women with PD. Moreover, available evidence remains inconsistent and is often based on small study populations. Therefore, the potential interactions between PD severity, body composition, and postural stability remain insufficiently understood.

Since recurrent pain episodes may induce longer-term neuromuscular adaptations, potential differences in postural control may remain detectable even outside the menstruation period. Therefore, the aim of this study was to investigate whether postural stability and body composition differ according to the severity of PD and whether menstrual pain intensity is associated with balance performance and body composition in young women. We hypothesized that postural stability and body composition may vary according to the severity of PD.

## 2 Materials and methods

### 2.1 Participant characteristics

This cross-sectional study was conducted at the Biomechanics Laboratory of the University Center for Research and Development in Health Sciences, University of Rzeszow, between January and February 2026. Approval to conduct the study was obtained from the Bioethics Committee of the University of Rzeszów (Resolution No. 2/01/2026).

Participants were recruited using a custom-designed, self-administered questionnaire designed to evaluate menstrual characteristics, pain severity, and general health status. Participants were included based on the following inclusion criteria: age between 18 and 28 years, regular menstrual cycles (28 ± 7 days), and provision of written informed consent. Exclusion criteria were as follows: age outside the defined range; any chronic neurological, orthopedic, psychiatric, or urogynecological disorders (other than PD); systemic diseases; visual or auditory impairments; vertigo; diarrhea on the day of testing; musculoskeletal injury within the 3 months preceding the study; use of medications affecting postural control or hormonal balance, including hormonal contraception and antidepressants; history of gynecological surgery; use of an intrauterine device; active menstruation on the day of testing; pregnancy (suspected or confirmed) or the postpartum period; and consumption of alcohol or psychoactive substances within 24 hours prior to the assessment (23, 24).

PD was identified based on self-reported recurrent menstrual pain and the absence of a previously diagnosed gynecological condition suggestive of secondary dysmenorrhea, as assessed using the study questionnaire.

A total of 128 women were initially invited to participate in the study. Participants were selected from among the candidates applying for admission to the University of Rzeszów for the 2025/2026 academic year. Following the application of inclusion and exclusion criteria, 99 participants were selected for further analysis. Based on their retrospective assessment of menstrual pain severity using the Visual Analogue Scale (VAS), participants were initially categorized into three groups: mild pain (VAS 1–3, n=19), moderate pain (VAS 4–7, n=58), and severe pain (VAS 8–10, n=22). The flow of participants throughout the study is presented in **Figure 1**.

**Figure 1:**
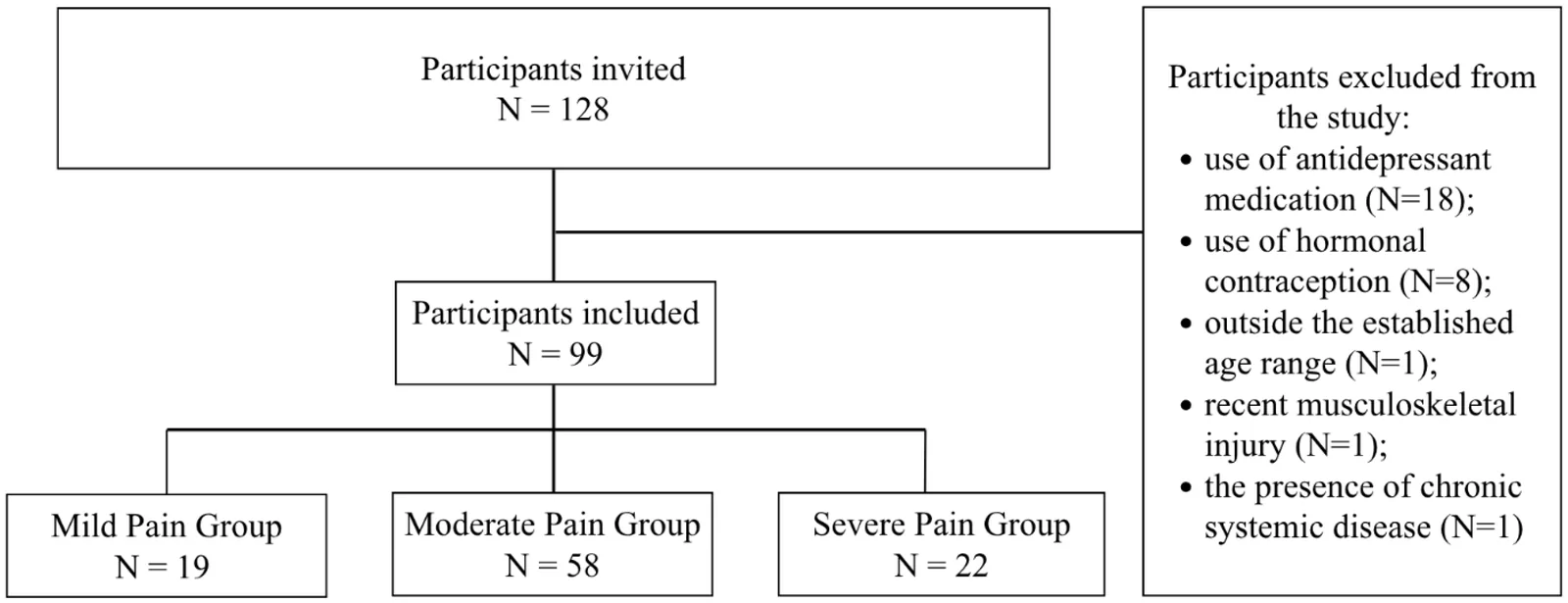
Flow diagram of patient disposition.

The required sample size for hypothesis testing was estimated *a priori* using G*Power software (version 3.1.9.7). Power analysis for comparing three independent groups, conducted at a significance level of α = 0.05 with an assumed power of 80% (1 – β = 0.80), indicated that a total sample size of 99 participants was required to detect a medium effect size (Cohen’s f ≈ 0.31).

All procedures were performed once. The study commenced with the completion of a custom-designed questionnaire, which was used to screen for the presence of study exclusion criteria. During this stage, information regarding symptoms associated with menstruation was also collected. The VAS was used for the subjective assessment of menstrual pain intensity. Subsequently, body composition was measured using a Tanita analyzer, followed by the assessment of static balance using the Neurocom platform.

### 2.2 Pain assessment

The intensity of menstrual pain was assessed retrospectively using the 10 cm Visual Analogue Scale. Participants were instructed to mark their pain intensity on a horizontal line, where 0 represents “no pain” and 10 represents “the worst imaginable pain” (25, 26). Pain intensity was evaluated specifically for the first two days of the menstrual cycle, coinciding with the period of peak prostaglandin concentration and the highest probability of symptomatic pain (27).

### 2.3 Body composition analysis

Body height was measured using a stadiometer (Charder, model MS 4900) with an accuracy of 0.5 cm, and these data were directly entered into the body composition analyzer to ensure measurement accuracy. Body composition was assessed using the Tanita body composition analyzer (model BC-420MA). To ensure standardization, measurements were performed in a single session under fasting conditions. Participants were instructed to remove outer clothing and electronic accessories, and measurements were taken in light underwear. Furthermore, individuals who had engaged in strenuous physical activity within the preceding hours were excluded from the analysis to minimize potential physiological bias (28, 29).

### 2.4 Balance assessment

Balance was assessed using the Neurocom SMART EquiTest system. During the assessment, participants stood barefoot in a standardized position, with feet parallel and centered on the dual force plates, and hands resting on their hips. Balance was evaluated using the Modified Clinical Test of Sensory Interaction on Balance (mCTSIB) protocol. This protocol involved assessment under four conditions (1): firm surface with eyes open (FIRM-EO) (2); firm surface with eyes closed (FIRM-EC) (3); unstable surface (using a dedicated 46 × 46 × 13 cm foam pad placed on the force plates) with eyes open (FOAM-EO); and (4) unstable surface with eyes closed (FOAM-EC). Each condition comprised three 30-second consecutive trials and the mean value of three trials was included in the final analysis (30).

### 2.5 Outcome measures

The primary outcome measure for statistical analysis was sway velocity of the center of gravity (COG) [degrees/second] obtained from NeuroCom SMART EquiTest system.

Secondary outcome measures included body composition parameters obtained using the Tanita BC-420MA analyzer: Percent of Body Fat (PBF) [%]; Fat Mass (FM) [kg]; Fat-Free Mass (FFM) [kg]; Muscle mass [kg]; Total Body Water (TBW) [kg]; Total Body Water (TBW) [% and kg]; and Bone Mass [kg].

### 2.6 Statistical analysis

Statistical analysis was performed using Statistica 13.3. Data normality was evaluated using the Shapiro-Wilk test. Continuous variables with normal distribution and homogenous variances (Levene’s test) were analyzed using one-way ANOVA; otherwise, the non-parametric Kruskal-Wallis test was applied. To evaluate the strength of the observed differences, an effect size measure was calculated for each comparison: Eta-squared (η²) for analyses of variance and Epsilon-squared (ϵ²) for rank-based analyses. If statistically significant differences had been detected (which was not the case in the final analysis), *post hoc* tests were planned (Tukey’s test for variance analyses and Dunn’s test with Bonferroni correction for rank-based analyses) to determine the specific differences between pairs of groups.

The correlations between subjective menstrual pain intensity, static balance indices, and body composition parameters were assessed using Spearman’s rank correlation coefficient (rs). The significance level for all statistical tests was set at α = 0.05.

## 3 Results

The study groups were comparable regarding demographic and anthropometric characteristics - no statistically significant differences were observed in terms of age (p = 0.926), body weight (p = 0.624), height (p = 0.842), or BMI (p = 0.673) (**Table 1**).

**Table 1:** General and physical characteristics of the participants.

Parameters		Statistics								
		N	*M*	*Me*	*Min.*	*Max.*	*Q1*	*Q3*	*SD*	*p*
Age [years]	Mild Pain	19	20,8	20,0	18,0	25,0	19,0	23,0	2,17	*H* = 0,15 0,926 ϵ²=0,00
	Moderate Pain	58	20,8	20,0	18,0	28,0	19,0	22,0	2,47	
	Severe Pain	22	20,5	20,5	18,0	25,0	19,0	21,0	1,95	
Body mass [kg]	Mild Pain	19	61,6	59,2	48,8	79,3	57,4	64,1	7,78	*H* = 0,94 0,624 ϵ²=0,01
	Moderate Pain	58	61,0	59,3	44,0	107,3	53,6	66,1	12,12	
	Severe Pain	22	62,6	59,3	47,9	83,8	54,5	68,8	11,15	
Height [cm]	Mild Pain	19	165,5	167,0	156,0	175,0	163,0	168,0	4,59	*F* = 0,17 0,842 η²=0,00
	Moderate Pain	58	165,5	165,5	152,0	181,0	162,2	170,0	5,81	
	Severe Pain	22	166,3	165,5	158,0	176,0	162,2	170,2	5,52	
BMI [kg/m^2^]	Mild Pain	19	22,5	22,2	17,7	29,8	20,5	23,0	3,02	*H* = 0,79 0,673 ϵ²=0,01
	Moderate Pain	58	22,4	21,6	16,4	39,4	19,4	23,5	4,29	
	Severe Pain	22	22,1	21,3	18,3	30,4	19,8	22,8	3,48	

N, number of observations; *M*, mean; *Me*, median; *Min*., minimum value, *Max*., maximum value; *Q1*, first quartile; *Q3*, third quartile; *SD*, standard deviation; BMI, Body Mass Index; *F*, statistic value (one-way ANOVA); *H*, Kruskal–Wallis statistic; *p*, *p-value.*

No statistically significant differences were observed between the study groups regarding menarche age (p = 0. 354), menstrual cycle length (p = 0. 468), or the duration of menstrual bleeding (p = 0. 705) (**Table 2**).

**Table 2:** Gynecological characteristics of the participants.

Parameters		Statistics								
		N	*M*	*Me*	*Min.*	*Max.*	*Q1*	*Q3*	*SD*	*p*
Age at menarche [years]	Mild Pain	19	12,4	12,0	11,0	15,0	12,0	13,0	1,17	*H* = 2,08 0,354 ϵ²=0,02
	Moderate Pain	58	12,8	13,0	11,0	16,0	12,0	13,0	1,37	
	Severe Pain	22	12,8	13,0	11,0	15,0	12,0	13,0	0,92	
Menstrual cycle length [days]	Mild Pain	19	29,2	28,0	25,0	35,0	28,0	29,5	2,46	*H* = 1,52 0,468 ϵ²=0,02
	Moderate Pain	58	29,2	29,0	21,0	35,0	28,0	30,0	2,77	
	Severe Pain	22	30,5	29,5	26,0	40,0	28,0	34,2	3,76	
Duration of menstrual bleeding [days]	Mild Pain	19	5,5	5,0	4,0	7,0	5,0	6,5	1,02	*H* = 0,70 0,705 ϵ²=0,01
	Moderate Pain	58	5,5	5,0	4,0	9,0	5,0	6,0	0,94	
	Severe Pain	22	5,8	5,0	4,0	8,0	5,0	6,8	1,23	

N, number of observations; *M*, mean; *Me*, median; *Min*., minimum value; *Max*., maximum value; *Q1*, first quartile; *Q3*, third quartile; *SD*, standard deviation; BMI, Body Mass Index; *H*, Kruskal–Wallis statistic; *p*, *p-value.*

Four trial variants from the mCTSIB protocol were analyzed. Statistical analysis revealed no significant differences in balance performance between groups with varying menstrual pain intensity in any of the conducted trials (all p > 0.05), with small effect sizes across comparisons (**Table 3**).

**Table 3:** A comparison of mean balance test scores (FIRM and FOAM) between groups with different severities of menstrual pain.

Parameter/Pain		Statistics								
		N	*M*	*Me*	*Min.*	*Max.*	*Q1*	*Q3*	*SD*	*p*
FIRM-EO Mean [deg/s]	Mild	19	0,35	0,33	0,20	0,53	0,27	0,42	0,10	*H* = 1,62 0,445 ϵ²=0,02
	Moderate	58	0,33	0,30	0,17	0,73	0,24	0,40	0,11	
	Severe	22	0,36	0,35	0,20	0,57	0,27	0,40	0,11	
FIRM-EC Mean [deg/s]	Mild	19	0,39	0,40	0,23	0,60	0,30	0,48	0,11	*H* = 3,17 0,205 ϵ²=0,03
	Moderate	58	0,35	0,33	0,17	0,80	0,27	0,42	0,11	
	Severe	22	0,35	0,33	0,20	0,67	0,27	0,40	0,11	
FOAM-EO Mean [deg/s]	Mild	19	0,80	0,80	0,53	1,00	0,68	0,90	0,14	*H* = 2,76 0,252 ϵ²=0,03
	Moderate	58	0,81	0,78	0,57	1,40	0,70	0,90	0,16	
	Severe	22	0,75	0,73	0,53	1,07	0,63	0,82	0,15	
FOAM-EC Mean [deg/s]	Mild	19	1,56	1,57	1,13	2,00	1,35	1,80	0,26	*H* = 2,36 0,307 ϵ²=0,02
	Moderate	58	1,56	1,58	0,97	2,73	1,30	1,75	0,34	
	Severe	22	1,47	1,50	0,97	2,47	1,28	1,58	0,33	

N, number of observations; *M*, mean; *Me*, median; *Min*., minimum value; *Max*., maximum value; *Q1*, first quartile; *Q3*, third quartile; *SD*, standard deviation; *H*, Kruskal–Wallis statistic; *p*, *p-value.*

Variables obtained via bioelectrical impedance analysis were used to assess differences in body composition parameters among the three identified groups. Statistical analysis revealed no significant differences for any of the examined body composition parameters, with small effect sizes across comparisons. The percentage of body fat was comparable across all subgroups (p = 0.746). A similar trend was observed for fat-free mass (p = 0.709) and muscle mass (p = 0.709). Total body water levels remained consistent across subgroups, ranging from 52,9% to 53,6% (p = 0.762) (**Table 4**).

**Table 4:** Comparison of body composition parameters across the study groups classified by pain severity.

Parameter/Pain		Statistics								
		N	*M*	*Me*	*Min.*	*Max.*	*Q1*	*Q3*	*SD*	*p*
FAT [%]	Mild	19	24,8	25,2	14,8	35,1	21,4	27,4	5,29	*H* = 0,59 0,746 ϵ²=0,01
	Moderate	58	24,1	23,4	3,0	47,6	19,0	28,7	8,22	
	Severe	22	23,3	23,4	3,0	37,8	15,6	28,9	8,90	
FAT-MASS [kg]	Mild	19	15,6	14,8	7,7	27,8	12,4	18,1	5,13	*H* = 0,61 0,735 ϵ²=0,01
	Moderate	58	15,6	13,6	1,3	51,1	9,9	18,1	8,94	
	Severe	22	15,1	14,0	1,7	30,8	8,5	19,7	7,97	
FFM [kg]	Mild	19	46,0	45,5	40,9	53,6	43,8	47,5	3,51	*H* = 0,69 0,709 ϵ²=0,01
	Moderate	58	45,6	45,5	39,2	61,0	42,4	48,1	4,07	
	Severe	22	46,4	45,8	40,8	54,6	42,9	48,9	3,85	
MUSCLE-MASS [kg]	Mild	19	43,6	43,2	38,8	50,9	41,6	45,1	3,34	*H* = 0,69 0,709 ϵ²=0,01
	Moderate	58	43,3	43,2	37,2	57,9	40,2	45,7	3,87	
	Severe	22	44,0	43,5	38,7	51,8	40,8	46,4	3,66	
TBW [kg]	Mild	19	32,4	32,1	28,5	38,1	30,7	33,5	2,73	*H* = 0,09 0,954 ϵ²=0,00
	Moderate	58	32,6	32,4	27,1	44,8	29,9	34,5	3,53	
	Severe	22	32,6	32,2	28,2	38,5	30,0	34,8	2,86	
TBW [%]	Mild	19	52,9	52,7	46,5	59,6	51,2	55,1	3,39	*H* = 0,54 0,762 ϵ²=0,01
	Moderate	58	53,6	54,0	38,6	80,0	50,6	56,7	5,97	
	Severe	22	53,3	53,8	44,8	60,6	50,5	57,1	4,79	
BONE-MASS [kg]	Mild	19	2,3	2,3	2,1	2,7	2,2	2,4	0,18	*H* = 0,80 0,670 ϵ²=0,01
	Moderate	58	2,3	2,3	2,0	3,1	2,2	2,4	0,20	
	Severe	22	2,4	2,3	2,1	2,8	2,2	2,5	0,20	

N, number of observations; *M*, mean; *Me*, median; *Min*., minimum value; *Max*., maximum value; *Q1*, first quartile; *Q3*, third quartile; *SD*, standard deviation; *H*, Kruskal–Wallis statistic; *p*, *p-value.*

A statistically significant negative correlation was observed in the mild dysmenorrhea subgroup during the FIRM-EC trial (r_s_ = -0.71; p < 0.001). This indicates that among women with the lowest pain intensity, an increase in VAS scores was associated with a substantial reduction in COG sway velocity. However, given the small subgroup size, this correlation should be considered exploratory and interpreted with caution. Conversely, no such association was observed in the moderate or severe dysmenorrhea subgroups (**Table 5**).

**Table 5:** Spearman’s rank correlation between pain levels on the VAS scale and the results of balance assessment.

Parameter	Pain level			
	Mild pain	Moderate pain	Severe pain	*Combined*
FIRM-EO Mean [deg/s]	*r_s_* = -0,21 *p* = 0,391	*r_s_* = -0,09 *p* = 0,520	*r_s_* = -0,20 *p* = 0,368	*r_s_* = -0,02 *p* = 0,865
FIRM-EC Mean [deg/s]	***r_s_* = -0,71 *p* < 0,001**	*r_s_* = -0,08 *p* = 0,529	*r_s_* = 0,18 *p* = 0,430	*r_s_* = -0,18 *p* = 0,078
FOAM-EO Mean [deg/s]	*r_s_* = -0,24 *p* = 0,316	*r_s_* = -0,07 *p* = 0,597	*r_s_* = -0,23 *p* = 0,294	*r_s_* = -0,16 *p* = 0,119
FOAM-EC Mean [deg/s]	*r_s_* = 0,10 *p* = 0,673	*r_s_* = 0,11 *p* = 0,416	*r_s_* = 0,18 *p* = 0,414	*r_s_* = -0,08 *p* = 0,430

*r_s_*, Spearman’s rank correlation coefficient; *p, p-value.* Bold values indicate statistically significant correlations (p<0.05).

The exact VAS scores for pain intensity did not correlate significantly (*p > 0.05*) with any of the body composition parameters, neither in the overall study population nor when stratified by pain severity (**Table 6**).

**Table 6:** Spearman’s rank correlation between pain levels on the VAS scale and body composition parameters.

Body composition parameter	Pain level			
	Mild	Moderate	Severe	*Combined*
FAT [%]	*r_s_* = -0,11 *p* = 0,654	*r_s_* = -0,14 *p* = 0,311	*r_s_* = 0,02 *p* = 0,927	*r_s_* = -0,11 *p* = 0,292
FAT-MASS [kg]	*r_s_* = -0,15 *p* = 0,544	*r_s_* = -0,13 *p* = 0,336	*r_s_* = 0,07 *p* = 0,757	*r_s_* = -0,10 *p* = 0,306
FAT FREE MASS [kg]	*r_s_* = -0,15 *p* = 0,552	*r_s_* = -0,07 *p* = 0,609	*r_s_* = -0,10 *p* = 0,667	*r_s_* = 0,00 *p* = 0,983
MUSCLE-MASS [kg]	*r_s_* = -0,15 *p* = 0,552	*r_s_* = -0,07 *p* = 0,606	*r_s_* = -0,10 *p* = 0,667	*r_s_* = 0,00 *p* = 0,982
TBW [kg]	*r_s_* = -0,13 *p* = 0,607	*r_s_* = -0,11 *p* = 0,369	*r_s_* = -0,10 *p* = 0,653	*r_s_* = -0,02 *p* = 0,838
TBW [%]	*r_s_* = 0,12 *p* = 0,626	*r_s_* = 0,14 *p* = 0,311	*r_s_* = 0,07 *p* = 0,742	*r_s_* = 0,09 *p* = 0,376
BONE-MASS [kg]	*r_s_* = -0,19 *p* = 0,440	*r_s_* = -0,04 *p* = 0,752	*r_s_* = -0,17 *p* = 0,449	*r_s_* = 0,03 *p* = 0,759

TBW, Total Body Water; *r_s_*, Spearman’s rank correlation coefficient; p, p-value.

## 4 Discussion

PD represents a pervasive health challenge among young women, a finding strongly corroborated by the data collected in the present study. Questionnaire-based assessments revealed that the vast majority of participants reported experiencing menstrual pain. Specifically, the moderate pain group comprised the largest portion of the cohort, followed by those reporting severe pain, while the mild pain group accounted for the smallest percentage of subjects. This distribution of pain severity within the study population is consistent with the results presented by Çınar et al., who observed an analogous prevalence pattern in their large-scale investigation of women with PD (31).

We hypothesized that PD severity may be associated with altered postural control and body composition; however, contrary to our hypothesis, our findings suggest preserved static postural control and no measurable body composition differences in young women with PD.

The analysis of the obtained results revealed no significant differences in static balance parameters across groups categorized by mild, moderate, and severe menstrual pain intensity. These findings may indicate that static postural control remains largely preserved despite recurrent menstrual pain. Moreover, effect sizes were consistently small across all balance comparisons, suggesting that any true differences, if present, are likely to be of limited clinical relevance. This observation is consistent with the findings reported by Mizuta et al., who noted the absence of statistically significant differences in static balance at the group level across various menstrual cycle phases. Notably, Mizuta et al. also demonstrated that individual symptom severity, particularly pain and concentration difficulties, correlates with variations in postural stability parameters, implying that the impact of menstrual pain on postural control is highly individualized rather than phase-dependent (32).

Regarding the disparities in body composition parameters across the established PD severity groups, the analysis revealed no significant differences in total body fat, visceral fat, or hydration levels. This lack of variation suggests that the participant groups were homogeneous, not only regarding BMI but also in terms of their overall somatic profile. Therefore, differences in body composition were unlikely to explain the observed balance outcomes in the present sample.

Another research objective involved evaluating the correlation between pain intensity, as assessed by the VAS, and balance parameters within the identified groups. Within the mild pain group, higher subjective menstrual pain intensity was associated with reduced center-of-gravity excursions during stable standing with eyes closed on the Neurocom platform. It can be hypothesized that women reporting slightly higher pain levels within the mild PD group may suggest a more rigid postural strategy, resulting in reduced sway velocity. However, in this study we did not directly assess muscle activation patterns and postural strategies.

No significant correlations were observed in the moderate and severe PD groups. This may indicate that the relationship between pain intensity and postural control is not linear and may be influenced by additional unmeasured factors. Alternatively, the observed association in the mild pain group may represent a subgroup-specific finding that requires confirmation in larger studies.

The results indicate no statistically significant relationship present between specific VAS pain scores and body composition parameters within the studied groups. This finding contrasts with recent evidence provided by Purwaningtyas et al., who reported a positive correlation between adipose tissue mass, pain intensity, and the duration of menstrual symptoms. The authors attribute this to the role of adipose tissue as a reservoir for arachidonic acid, a primary substrate for the synthesis of proinflammatory prostaglandins that induce pain. However, it should be noted that the population examined by Purwaningtyas et al. was highly heterogeneous, whereas the present study analyzed a cohort standardized by BMI. In such a homogenous sample, minor fluctuations in adipose tissue were not associated with the severity of reported pain in the present sample (33).

The present study employs an interdisciplinary framework by integrating gynecology, physiotherapy, and biomechanics. To our knowledge, few studies have simultaneously examined PD severity, body composition, and objective postural stability. By combining objective postural control assessment with body composition analysis, this research moves beyond traditional gynecological models to explore the impact of PD on overall motor function. A notable strength of this study is the data collection during the intermenstrual phase, which allowed for body composition analysis and successfully isolated underlying postural status from acute, pain induced reactions. Although global balance deficits were not identified, this methodological design facilitated the detection of subtle compensatory mechanisms.

Despite its methodological strengths, this study has inherent limitations. Firstly, the study involves a relatively small sample size and a short duration, which limits the broader generalizability of the findings. Furthermore, it lacked standardized psychometric assessments for anxiety, kinesiophobia, or catastrophizing, which would have enabled a clearer distinction between the physiological and emotional components of pain. Also, differences in habitual physical activity were not controlled. Additionally, the assessment of gynecological health relied primarily on self-reported questionnaires regarding medical history rather than direct clinical evaluations, such as transabdominal or transvaginal ultrasound examinations or physical examinations performed by a gynecologist. Secondly, while the VAS scale is a standard clinical tool, it captures subjective pain intensity rather than objective stimulus magnitude, making results dependent on individual pain tolerance. Moreover, the study is limited by the lack of direct biochemical markers to assess hormonal status, which restricts deeper endocrinological insights into the mechanisms underlying PD. Additionally, although measurements were strictly conducted outside the menstruation window, the lack of standardization regarding the specific menstrual cycle phase could introduce variability. Finally, the study’s cross-sectional design relied on single inter-menstrual measurements and retrospective pain reports, which may be subject to recall bias. Therefore, future longitudinal research, involving repeated assessments of the same subjects across multiple cycle phases, is recommended to better capture the true dynamics of postural adaptation.

### 4.1 Conclusions

In this sample of young women, PD severity was not associated with differences in static postural stability or body composition parameters. A significant association between pain intensity and sway velocity was observed only in the mild PD subgroup and should be interpreted cautiously until confirmed by future studies. These findings suggest that static postural control remains largely preserved across different levels of PD severity during the inter-menstrual period.

## Data Availability

The original contributions presented in the study are included in the article/supplementary material. Further inquiries can be directed to the corresponding author.
